# Application of ChatGPT in pediatric surgery: opportunities and challenges

**DOI:** 10.1097/JS9.0000000000001195

**Published:** 2024-02-07

**Authors:** Changkun Mao, Yuanyuan Bao, Yuanyuan Yang, Yongsheng Cao

**Affiliations:** a Department of Urology, Anhui Provincial Children’s Hospital; b Department of Electrocardiogram, Anhui Maternal and Child Health Hospital, Hefei, Anhui, People’s Republic of China

With the rapid development of artificial intelligence (AI) technology, large language models like Chat Generative Pre-trained Transformer (ChatGPT) are increasingly being applied in various fields. Particularly in the field of medicine, they offer unprecedented assistance to healthcare services^1^. Pediatric surgery, as a crucial branch of medicine, stands to benefit from these technologies, yet it also faces unique challenges. This article aims to explore the potential applications of ChatGPT in pediatric surgery, along with the opportunities and challenges that accompany its use.

In the realm of pediatric surgical disease diagnosis, ChatGPT can provide significant assistance. Through deep learning and the analysis of extensive medical data, ChatGPT can aid physicians in identifying specific disease patterns and symptoms, particularly excelling in early diagnosis^2^. Additionally, it can integrate clinical symptoms, laboratory test results, and imaging data to provide a more comprehensive overview of diseases. This analytical capability of ChatGPT is particularly crucial in dealing with unique disease manifestations in children, where symptoms may significantly differ from those in adults, and traditional diagnostic methods may be less sensitive or limited. Furthermore, ChatGPT can offer personalized treatment recommendations based on specific patient information, such as age, gender, and genetic background, crucial for enhancing treatment effectiveness and predicting disease progression. In emergency situations, such as acute infections or trauma, ChatGPT’s rapid response capability can provide immediate support to physicians’ decision-making, which is especially crucial in emergency and critical care medicine^3^. Importantly, by offering data-driven recommendations, ChatGPT helps reduce diagnostic errors, especially when physicians face high workloads or lack experience.

Pediatric patients often require special communication and educational approaches, where ChatGPT plays a crucial role in enhancing patient communication and education. Due to the distinct understanding, emotional needs, and communication styles of pediatric patients compared to adults, traditional healthcare communication methods may fall short of meeting their unique requirements. In such cases, ChatGPT, with its highly adaptive and interactive communication, can effectively connect with pediatric patients and their families. It can use simple, understandable language to explain complex medical information, aiding children in understanding their conditions and treatment processes, which is crucial for reducing anxiety and improving treatment compliance. Moreover, ChatGPT can incorporate interactive educational materials, such as games, stories, and animations, to engage children’s attention, making health education more interesting and effective. This approach is particularly useful for children undergoing surgical treatment, such as educating them on how to relax before entering the operating room, thereby minimizing stress and crying. Through these means, ChatGPT not only provides information but also stimulates children’s curiosity and learning interests, having a long-term positive impact on promoting children’s responsibility for their health. Importantly, ChatGPT can offer personalized educational content, adjusting the presentation of information based on the child’s age, interests, and comprehension level, enhancing the effectiveness of education. In summary, ChatGPT plays a significant role in strengthening communication and education for pediatric surgical patients, innovatively improving interaction between physicians and pediatric patients, and making health education more effective and appealing.

The application of ChatGPT in pediatric surgery also presents challenges, particularly in data privacy and security. Dealing with sensitive information of pediatric patients, including personal health information, family medical history, and genetic data, poses complex and critical issues. The protection of this information is paramount for maintaining patient privacy. When using ChatGPT to process this information, it is imperative to ensure compliance with relevant data protection regulations such as the Children’s Online Privacy Protection Act (COPPA) and the Health Insurance Portability and Accountability Act (HIPAA). This requires a rigorous review of any patient data use, ensuring data security and anonymity. Similarly, the use of AI in pediatric healthcare raises a range of ethical issues^4^. Firstly, the consent and participation rights of pediatric patients need consideration. As a special population with limited understanding and ability to express consent, healthcare professionals and technology developers must exercise caution when using AI technology. Additionally, transparency and fairness in AI decision-making must be considered to avoid diagnostic and treatment inequalities resulting from data biases. Balancing algorithm transparency with commercial confidentiality is also a matter of concern.

Despite the excellent performance of ChatGPT in processing medical information, concerns about its accuracy and reliability persist in certain situations^5^. The accuracy and reliability of ChatGPT heavily depend on the quality and representativeness of input data. If the data used are biased, incomplete, or outdated, the diagnostic and treatment recommendations proposed by the model may be inaccurate. This is particularly important in pediatric surgery, where the symptoms and responses of pediatric patients may differ from those of adults, and data for certain pediatric diseases may be scarce. For complex or rare pediatric diseases, ChatGPT may struggle to provide accurate diagnostic and treatment recommendations due to the limited number of clinical cases, and the highly personalized nature of symptoms and treatment responses. Moreover, as medicine is a continually advancing field, keeping the ChatGPT model updated and synchronized with the latest medical developments is a significant challenge. If the model is not updated promptly, the information it provides may become outdated, impacting its reliability. There is also a risk of healthcare professionals or other medical personnel excessively relying on the information provided by ChatGPT, potentially overlooking the specific circumstances of patients and the professional judgment of doctors, affecting the reliability of treatment. Therefore, there are many concerns and objections to the use of AI, especially in medicine, where medicine is supposed to treat and save people’s lives.

In conclusion, the application of ChatGPT in pediatric surgery demonstrates significant potential, assisting in diagnosis, patient education, and accelerating clinical research. However, the accompanying changes in data security, ethics, accuracy, and doctor–patient relationships pose new challenges. Moving forward, the application of ChatGPT in pediatric surgery is poised to evolve, shaping the future landscape of healthcare for children. The potential for enhanced accuracy, efficiency, and improved patient experiences opens doors to further exploration and innovation. Discussing future visions and potential research directions in integrating ChatGPT into pediatric surgical practice becomes paramount. The ethical considerations, data security measures, and ongoing advancements in AI technology will continue to influence and guide the responsible application of ChatGPT in pediatric surgery. As we embark on this transformative journey, collaborative efforts among healthcare professionals, technology developers, and policymakers will be essential to unlock the full potential of AI in pediatric surgical care.

## Ethical approval

Not applicable.

## Consent

Not applicable.

## Source of funding

Not applicable.

## Author contribution

C.M.: conceptualization and writing; Y.B.: design and conceptualization; Y.Y. and Y.C.: review, editing, and supervision.

## Conflicts of interest disclosure

The authors declare no conflicts of interest.

## Research registration unique identifying number (UIN)

Not applicable.

## Guarantor

All authors.

## Data availability statement

Data are available from the corresponding author if justification for the requirement is provided.

## Provenance and peer review

Yes, we agree.
